# Effect of Baby Walker Use on Developmental Status based on Ages and Stages Questionnaire Score (ASQ)

**Published:** 2020

**Authors:** Omid YAGHINI, Mehrdad GOODARZI, Samin KHOEI, Mehrnoosh SHIRANI

**Affiliations:** 1 Child Growth and Development Research Center; School of Medicine; Isfahan University of Medical Sciences; Isfahan; Iran Email: yaghini@med.mui.ac.i

**Keywords:** Baby walker, Infant, Ages and stages questionnaire, Development

## Abstract

**Objectives:**

Baby walker is a popular device, which parents use for entertainment, keeping infants safe and walking promotion. We aimed to determine whether baby walker usage has any effect on the development using Ages and Stages Questionnaire (ASQ).

**Materials & Methods:**

We evaluated 107 one-yr-old infants in each baby walker user group and non-users (214 participants) using ASQ test of 12-month in Isfahan health centers for vaccination in 2017. We re-evaluated 168 infants at the age of 18-month using ASQ test of 18-month. The data of these groups were compared.

**Results:**

Girls use baby walker more frequently (*P*=0.02). Baby walker usage was not significantly associated with parent’s educational state, mother employment, birth rank of infant and delivery method. The starting age of baby walker use was 6.61 ± 1.46 months. ASQ results in area of gross movement and fine movement were not significantly different in users and non- users at age 12 and 18 months.

**Conclusion:**

Most parents believe baby walker can promote earlier walking, but based on current evidence this belief might not be true. Although most studies showed no developmental delay in baby walker users, parents should become aware of their possible negative effects and hazards.

## Introduction

Baby walker is a popular device on which a lot of money is spent annually (1). Baby walker usage reported 64%–92% in different population (2-4). In Tehran, Iran, 54.5% of infants used baby walker and many parents believe that using baby walker helps their infants to walk earlier (5). In addition to possible negative effects on development, there are many concerns about baby walker-related injuries. About 12%-40% of baby walker users have experienced at least one related injury. Because of these possible side effects and lack of evidence to support benefits of baby walker usage the American Academy of pediatrics recommends prohibition of the production and purchase of baby walkers (2). 

The studies conducted to show whether baby walker use has significant effect on the development have led to conflicting results. Some studies reported no significant difference in gait acquisition between baby walker user and non-user infants (6-8); while others reported delayed motor development (9, 10). Only one study showed significantly earlier walking skills in the user group (11). These studies are not reliable enough due to limitations including sample size, failure to randomize and absence of control group (12). Moreover, none of them used Ages and Stages Questionnaire (ASQ), which is one of the validated tests for evaluation of developmental status in Iran (13). Hence their results cannot be generalized to the Iranian population.

We aimed to determine the effect of baby walker on the developmental milestones in ASQ test on one-yr-old infants in Isfahan, central Iran. 

## Materials & Methods

This cross-sectional study was performed on one-yr-old infants referred to Isfahan health centers for vaccination in 2017. Three centers were selected by clustering method, 220 one-yr-old infants were entered in the study by easy sampling. 

Informed consent was taken from parents of the infants participated in this study. Ethics Committee and Pediatrics Review Board of Isfahan University of Medical Sciences approved the protocol of this study.

Exclusion criteria were preterm delivery, existence of congenital neurodevelopmental disorders and obvious developmental disorders before using baby walker. Six infants were excluded from the study accordingly.

We collected 107 infants in each baby walker user and non-user group. ASQ test of 12-month and questionnaire papers were filled for all infants. We re-evaluated 168 infants (81 in user and 87 in non- user group) at the age of 18 month by ASQ test of 18-month.

**Table 1:** Demographic characteristics of sample

Parameter		Baby walker user		Non baby walker user		Test used	Test value	*P*-value
		Number	%	Number	%	-	-	-
infants		107	50	107	50			
sex	boy	41	19.2	57	26.6	Chi square	4.81	0.02
	girl	66	30.8	50	23.4			
Maternal education	High school	41	19.2	48	22.4	Exact fisher	1.28	0.52
	Diploma to bachelor	64	29.9	56	26.2			
	Master to doctoral	2	0.93	3	1.4			
Paternal education	High school	40	18.9	48	22.5	Chi square	1.95	0.37
	Diploma to bachelor	57	26.9	48	22.5			
	Master to doctoral	8	3.8	11	5.2			
Mother employment	employed	30	14	24	11.2	Chi square	0.34	0.43
	Unemployed	77	36	83	38.8			
Birth rank	1st	50	23.4	37	17.3	Chi square	6.43	0.38
	2nd	36	16.8	39	18.2			
	3rd	16	7.4	22	10.3			
	4^th^ and <	5	2.3	9	4.2			

**Table 2:** Comparison of weight and head circumference between baby walker users and non-users

Parameters	Users Average ± Standard deviation	Non-users Average ± Standard deviation	Test	Test value	*P*-value
Birth child weight (kg)	3.19±0.35	3.20±0.38	Independent T	0.29	0.77
12 Month Weight (kg)	9.42±0.62	9.51±0.74	Independent T	0.84	0.39
Birth head circumference (cm)	34.66±1.04	34.76±1.15	Mann-Whitney	0.83	0.40
12 month circumference (cm)	46.05±1.04	46.11±1.37	Mann-Whitney	0.06	0.94

**Table 3:** Comparison of ASQ test results between baby walker users and non-users

Parameter		User		Non user		Test	Test value	*P*-value
		N	%	N	%			
12 month gross motor	-1SD <	104	48.6	106	49.5	Exact fisher	1.09	0.31
	-2SD < ≤-1SD	3	1.5	1	0.5			
	≤-2SD	0	0	0	0			
18 month gross motor	-1SD <	80	47.6	86	51.2	Exact fisher	003/0	00/1
	-2SD < ≤-1SD	1	0.6	1	0.6			
	≤-2SD	0	0	0	0			
12 month fine motor	-1SD <	107	50	105	49.1	Exact fisher	2.01	0.49
	-2SD < ≤-1SD	0	0	1	0.5			
	≤-2SD	0	0	1	0.5			
18 month fine motor	-1SD <	78	46.4	87	51.8	Exact fisher	3.28	0.11
	-2SD < ≤-1SD	3	1.8	0	0			
	≤-2SD	0	0	0	0			

Questionnaire sheet included infant sex, age and educational state of parents (high school, diploma to bachelor, master to doctoral), employment state of mother, number of children in family, delivery method (normal vaginal delivery or caesarian section), gestational age at birth (term, preterm), birth and 12-month weight, birth and 12-month head circumference, starting age of baby walker use and parent’s purpose of baby walker use (to promote walking, entertainment, tradition)

Information was evaluated using EXCEL software version 2010 and SPSS version 23 (Chicago, IL, USA) and at a significant level of 0.05. The Mann-Whitney and Exact Fisher test was used to compare the developmental status in Gross motor and Fine motor areas (normal, one standard deviation and two standard deviations from normal. This project approved in ethical committee of IUMS (aproval code:ir. Mui. Rec. 1395.3.443).

## Results

Overall, 214 one-yr-old infants were enrolled. Most infants (54%) were girls. Among parents, 56% of mothers and 49% of fathers had educational degree from diploma to bachelor. Overall 74.8% of mothers were unemployed (Table 1). The mean age of mothers was 28.4±4.82 yr in user group and 29.5±4.57 in non-users (*P*=0.9). The mean age of fathers was 32.46±5.11 yr in users and 33.54±5.5 in non-user group (*P*=0.18). The mean birth weight was 3.20±0.37 kg and the mean birth head circumference was 34.71±1.10 cm (Table 2). 

Among our samples 51.4% of infants in user group and 49.5% infants in non-user group were born with normal vaginal delivery (*P*=0.78).

The starting age of baby walker use was 6.61±1.46 months in the group of users. Among parents of baby walker users, 56% believed that baby walker helps infant to walk earlier, 33% were using baby walker to keep infants occupied and 11% were using baby walkers for other reasons. 

Among baby walker users, 2.8% and 1.2% of infants had abnormal result in ASQ in the area of gross movement in 12 and 18 months of age, respectively. This proportion was 0.9% and 1.1% in non-user group at 12 and 18 months of age respectively (Table 3).

In the area of fine movement, all the infants in user group had normal ASQ result although 0.93% of non-users had abnormal result at age 12 months (*P*=0.49). At the age of 18-month fine movement abnormality was found in 2.8% of the users but the entire group of non-users had normal result (*P*=0.11).

## Discussion

According to our results, although gross movement abnormality was more frequent in baby walker users at age 12 month, no significant difference was found between the two groups in the area of ​​gross movements at the age of 12 and 18 months (*P*=0.31 and *P*=1, respectively). Data analysis showed no significant difference in the area of fine movement, social skills and language of ASQ test between users and non-users. Although baby walker usage was significantly higher in girls, there was no relationship between the use of baby walker and maternal employment, delivery method and educational state of parents. Baby walker usage was higher in employed mothers, infants born by caesarian delivery and in mothers with higher education (5). In Kashan, Iran, baby walker usage was not significantly higher in infants of employed mother (14). 

By the time we started this research, no article had been published on the relationship of ASQ test result and baby walker usage in literature. Other similar studies performed have also led to conflicting results. In two study crawling and walking independently occurred significantly later in the baby walker user group (9, 10). In a baby walker group, a significant delay was reported in the acquisition of all motor skills (14). Baby walker users also achieved lower scores in Bayley test compared with non-user group in Siegel study (9). On the other hand, despite the delayed onset of crawling in the baby walker user group, there was no significant difference in the onset of independent walking between the users and non-users (15). Four other studies also showed no difference on the age of gait acquisition between these two groups (5-8). Contrarily baby walker user infants had earlier walking skills compared with non-users. Although the two groups had no significant difference in motor development using AIMS(11).

There were some limitations in our study. We did not determine baby walker usage prevalence. We could not collect any information about baby walker-related injuries due to time limitation and lack of reliable information in health care system. Many factors can affect neurodevelopment such as congenital infections, congenital anomalies, hypothyroidism, labor complications, economic and educational state of family and substance use during pregnancy (16, 17). We tried to control the effect of these extraneous variables by defining exclusion criteria and random sampling, but they may still have affected the outcome. The percentage of abnormal ASQ result in our study was lower than other studies in Iran (18). This might be due to exclusion of infants with obvious developmental delay before using baby walker and infants with congenital neurodevelopmental problems, different target population, and data collecting errors.

Most parents believe that using baby walker promotes earlier walking in their infants (5, 19); and in many cases, parents use baby walkers to keep infants safe (19). 

**In conclusion,** much more studies should be carried out on this subject to accept or reject this belief. Current evidence are not enough to ban usage of baby walker, but parents should become aware of their possible negative effects and hazards. 
